# Global prevalence of anemia in displaced and refugee children: A comprehensive systematic review and meta-analysis

**DOI:** 10.1371/journal.pone.0312905

**Published:** 2024-11-22

**Authors:** Bisrat Birke Teketelew, Elias Chane, Abiy Ayele Angelo, Mebratu Tamir, Negesse Cherie, Mesele Nigus, Zewudu Mulatie, Dereje Mengesha Berta

**Affiliations:** 1 Department of Hematology and Immunohematology, School of Biomedical and Laboratory Sciences, College of Medicine and Health Sciences, University of Gondar, Gondar, Ethiopia; 2 Department of Clinical Chemistry, School of Biomedical and Laboratory Sciences, College of Medicine and Health Sciences, University of Gondar, Gondar, Ethiopia; 3 Department of Immunology And Molecular Biology, School of Biomedical and Laboratory Sciences, College of Medicine and Health Sciences, University of Gondar, Gondar, Ethiopia; 4 Department of Medical Parasitology, School of Biomedical and Laboratory Sciences, College of Medicine and Health Sciences, University of Gondar, Gondar, Ethiopia; 5 Department of Quality Assurance and Laboratory Management, School of Biomedical and Laboratory Sciences, College of Medicine and Health Sciences, University of Gondar, Gondar, Ethiopia; 6 Department of Hematology & Immunohematology, Wollo University, Wollo, Ethiopia; Università degli Studi di Milano, ITALY

## Abstract

**Background:**

Anemia due to living condition disproportionally affects the global refugee children. Nutritional deficiency particularly iron deficiency is the primary causes of anemia. Hence, we conducted a systemic review and meta-analysis on the prevalence of anemia among refugee children.

**Methods:**

We searched systematically all relevant studies on the prevalence of anemia among refugee children including under the age of 18 years, which were conducted between 2009 and 2023 in English from PubMed, Embase, Scopus, Cochran library and other gray literatures such as google scholar. Two researchers independently screened articles based on their title and abstract. The Joana Brigg’s Institute (JBI) checklist was used to assess the quality of studies. Random effect model was utilized to calculate the pooled estimate of anemia. Higgins I^2^ statistics and Egger’s test were analyzed to check heterogeneity and publication bias, respectively. Subgroup analysis by continent, age group of the children and year of study was employed to identify the source of heterogeneity.

**Result:**

A total of 14 studies were included in the final meta-analysis. Most of the studies were from Asian countries followed by African countries. The pooled prevalence of anemia among global refugee children was 36.54 with (95% CI: 23.79, 49.28). There was moderate level of heterogeneity between the studies (I^2^ = 68.91, P<0.001). The highest pooled prevalence of anemia in refugee children was reported in African, it was 56.1%, whereas the lowest pooled prevalence of anemia was in N. America, it was 12.66%. The prevalence of anemia was highest (53.88) in studies done among under-five refugee children.

**Conclusion:**

The global prevalence of anemia among refugee children is found to be moderate public health problem. Anemia prevalence is more common in African refugee children. Intervention and prevention for should be focused especially for refugees found in low- and middle-income countries.

## Background

People are fled from their permanent resident area, even cross the border and reside in different refugee countries [1]. Refugees in the glob reaches to high due to different disasters. Currently, the disasters particularly conflicts around the world rise challenging social, political, economic and humanitarian crisis. By the mid of 2023, global displacement reaches 110 million and 75% of is from low and middle income countries [2]. As a result, they are suffering from both communicable and non-communicable diseases [3]. Anemia is one of the most common problems in refugee setting and needs a special concern throughout the world with special focus on refugee and displacement settings [4].

Anemia is a global public health problem affecting one third of the world’s population and contributed to mortality, morbidity, impaired neurological development in children and decreased work productivity in adult [5]. Clinically, anemia is defined as a decreased in Red Blood Cell (RBC) number, Hemoglobin (Hb) concentration and volume occupied by RBCs or hematocrit of an individual. It can be classified in different categories based on the pathophysiology or morphological classification. Based on the morphological classification, there are three common types of anemia including; microcytic, normocytic and macrocytic anemia. The most common type of anemia is microcytic anemia due to iron deficiency [6]. Iron deficiency anemia (IDA) accounts 50% of the cause of anemia with a significant health consequence. IDA affects the cognitive function, academic and work performance of individuals [7]. Children are more vulnerable groups of anemia specifically due to iron deficiency. In children IDA can result in developmental delay and behavioral disturbance. The high onset of anemia in children and women is due to increased need during growth in children and increased iron demand in pregnancy [8].

Globally, 40% of children are suffering from anemia and most are from resource constrained countries. In developing country particularly in Africa there is high prevalence of anemia in children (60.2%) [9]. The lowest prevalence was observed in Rwanda (7) while highest prevalence in Senegal (87%). The burden of anemia contributes the greatest hospitalization in Sub-Saharan countries with estimated prevalence of 67% and significant fatality rate (6–18%) [10]. The increase in anemia prevalence in such resource constrained countries is without the counter balance of feeding practice [11]. The impact of anemia becomes high where people living in displaced and refugee settings particularly in children [4]. Like the general population, there was high prevalence of anemia reported in refugees and displaced children from resource limited countries such as 84% in Cameroon and in highly conflict area like Palestinian 59.7% [12, 13].

Nutritional deficiencies, chronic diseases and infections are generally the most common contributing factors for anemia in refugees. The effect of acute malnutrition and anemia in refugee children is high [14, 15]. This is due to inadequate iron intake and other nutritional deficiencies, and limited access to healthcare facilities. Lak of appropriate and complementary food, a high rate of infection due to overcrowding in refugee are also contributors for the high onset of anemia. For instance, if children left untreated to infection and infestation particularly for parasites, they have a high chance to have anemia. Because, most parasites live in the gastrointestinal tract (GIT) by feeding and competing of nutrients which limit the available micronutrient for cellular formation and development. Here, iron is the most common affected nutrient during parasite infection, consequently, limits the red blood cell development later results in anemia [16–18].

Studies were conducted and showed the prevalence of anemia in different part of the globe [4, 19–22]. As the reports showed, anemia in refugee and displaced children was high with variable magnitude in different countries. Therefore, it needs comprehensive data concerning with anemia prevalence in refugee and displaced children which is crucial due to the high vulnerability and unique challenges faced by these population. Conducting systemic review and meta-analysis provides pooled estimate on the prevalence, and information on the cause and possible intervention measures of anemia in refugee setting with particular focus on children, thereby informing targeted strategies and policies. It is important that policy makers and other stockholders possibly utilize the reliable data to reduce the burden of anemia in displaced and refugee settings. Moreover, this evidence-based information is important in improving the overall health outcomes, reducing morbidity and increasing the overall well-beings and quality of life in refugee and displaced children. The impact of various factors on the prevalence of anemia in refugee children were also systematically reviewed. Considering this, policy makers and other aid organizations can access reliable information and provides possible solutions for reducing the prevalence of anemia by preventing the underlying causes.

## Methods

### Study design

The Preferred Reporting Items for Systematic Reviews and Meta-Analyses (PRISMA) guideline was used and performed for this systematic review and meta-analysis. This review was registered on PROSPERO with registration number of CRD42024531837.

### Review questions

Based on the PRISMA guideline we explicit some review questions on anemia prevalence in global refugee children by focusing on some key components that aligned with systemic review process.

- What is the global distribution of anemia prevalence in refugee children aged 0–18 years and what risk factors may affect the onset of anemia?
- Are there differences in anemia prevalence between refugee children from different countries of origin?

### Eligibility criteria

#### Inclusion criteria

We included studies revealed on the prevalence of anemia at refugee camp or displaced children and resides at refugee all over the world and cross-sectional studies with available full text, and published in English language, between 2009 and 2023 were included.

#### Exclusion criteria

Studies with incomplete information such as not reported the sample size, studies with too small sample size as it didn’t represent the source population, the prevalence of anemia, without full text articles or studies with not clear study methodology, surveillance studies without reported clear sample size, insufficient and ambiguous data source for the meta-analysis process, case reports, review articles, poster presentations and editorial letters were excluded from the study.

### Data sources and searches

A systematic literature search using PubMed, Scopus, Embase, and Cochrane library was done up to January 23/2024 to obtain studies showing the prevalence of anemia in refugee and displaced children. The search was done in English language. An example of search term was as follows:

(((((prevalence) OR (magnitude)) OR (burden)) AND ((anemia) OR (iron deficiency anemia))) AND ((((((displacement) OR (refugee)) OR (refugee camp)) OR (evacuee)) OR (fugitive)) OR (expatriate))) AND ((children) OR (under five children))

We have also extended our searches by including other databases and grey literatures such as google scholar and African Journal Online (AJOL). Further research was found by a manual search as well as through the references listed in the relevant articles.

## Selection process

After carefully searching using the search term, articles were exported to EndNote x7 and the selection process was made on this software to manage search results and remove duplicates. Based on the inclusion criteria two researchers (BB Teketelew and DM Berta) had independently screened articles based on their title and abstract. These two researchers also screened the full text of articles weather it meets the inclusion criteria. At the same time the third researcher (Angelo AA.) carefully view and resolved the disagreement between the former researchers.

### Methodological quality assessment

Joanna Briggs Institute (JBI) critical appraisal checklist for the prevalence studies was used to evaluate the methodological quality of included studies [6]. The included studies were independently evaluated with nine methodological items of the checklist by two researchers (BB Teketelew and DM Berta). Based on the sum of the nine-quality item the articles were considered as low risk of bias with a high-quality score. Researchers then discuss on the final fate of the articles, and studies >50% were included in the final analysis. Moreover, the missing data was handled by excluding studies from the meta-analysis due the difficulties to impute such critical data.

### Data abstraction

All relevant data were extracted independently using Microsoft excel data extraction format. In the data extraction format, we included, first author name, year of publication, country, continent, study period, study design, sample size, age range of the study participants and prevalence of anemia in refugee or IDP children.

### Statistical analysis

After extracting data using a Microsoft Excel, it was exported to Stata software Version 17.0 for analysis and management. The standard error of prevalence for each original article were first calculated and then the study-specific estimates were pooled through a random-effects meta-analysis model to obtain an overall summary estimate of anemia prevalence. Text, tables, graphs and forest plot were used to present the data. The heterogeneity of the previous studies was assessed using I ^2^ (%) tests and the value 70% and over were considered as high heterogeneity. Random and fixed effect model were analyzed for high heterogeneity and low heterogeneity studies, respectively. Subgroup analysis was employed to assess the possible cause oh heterogenicity. Egger’s test was performed to check the presence of publication bias.

## Result

**Fig 1:**
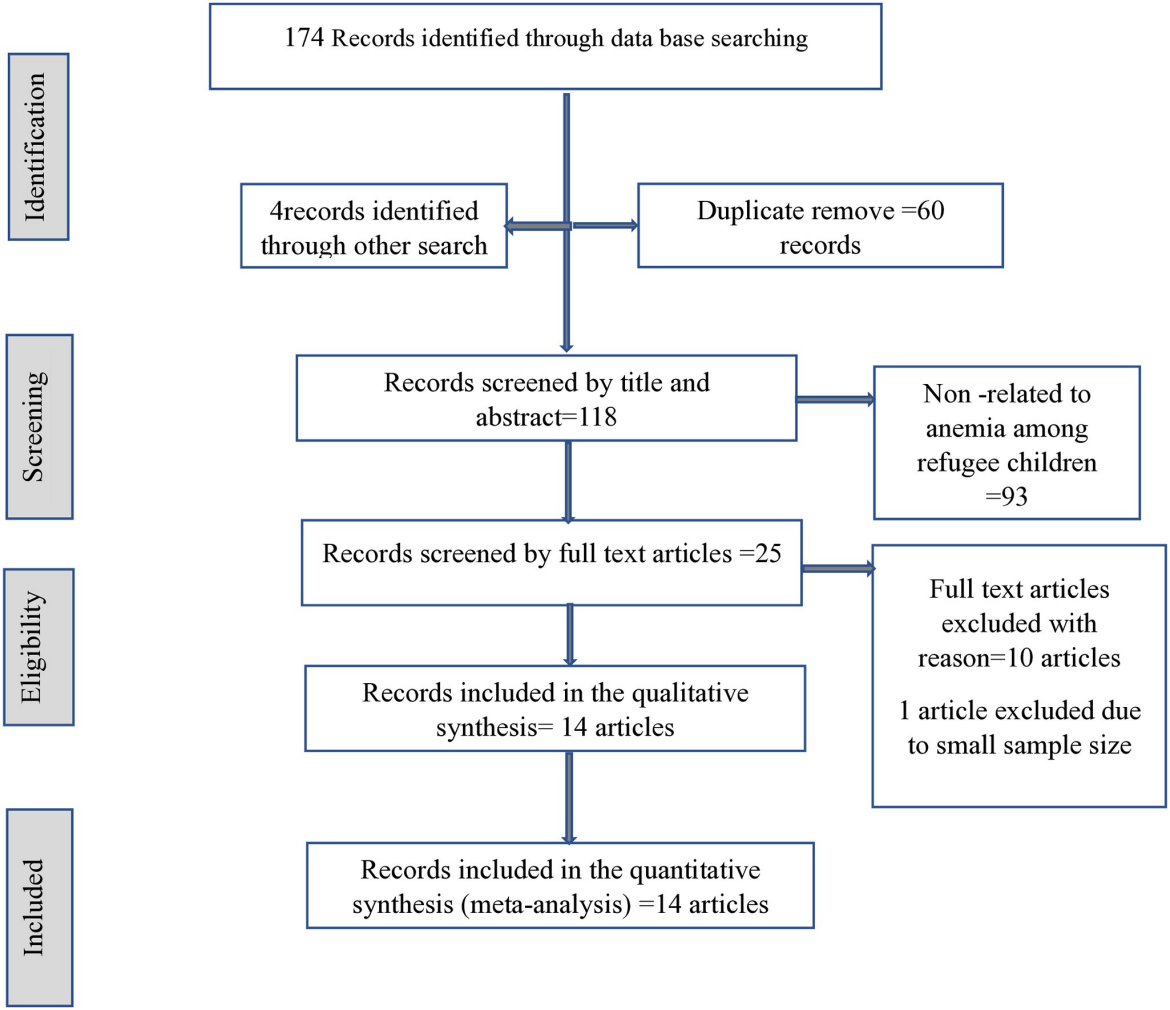
Study selection profile.

A total of 178 articles were first retrieved (174 from data base searching while 4 of them from other searches). Due to duplication 58 articles were removed. further 114 articles were screened by their title and abstract the 23 full text articles were screened. Finally, a total of 14 articles were included for the meta-analysis Fig 1.

## Characteristics of the included studies

A total of 14 studies which assessed the prevalence of anemia among refugee children were included in the final meta-analysis. Of included studies, most of were from Asian country [13, 23–25] followed by African country [26–30]. The smallest sample size in the included study was 215, which was from Syria conducted by Theresa et.al, [25]. The highest prevalence of anemia in refugee children was reported in in Cameroon from Africa, which was 84%, while the smallest prevalence was from Toronto (11%) Table 1.

**Table 1:** Characteristics of studies included in the meta-analysis of prevalence of anemia among refugee children.

First authors, year	Continent	Country	Study design	Age range	Sample size	prevalence%
Carolyn Beukeboom, 2018, [31]	N. America	Canada	retrospective	0–16 years	356	15.7
Vanessa J. Redditt, 2015 [32]		Toronto	retrospective	0–15 years	1063	11.0
Ankoor Y. Shah, 2013 [33]	Europe	Georgia	retrospective	0–18 years	555	17.7
Ioanna D. Pavlopoulou, 2017, [34]		Greece	cross sectional	1–14 years	300	13.7
Joana Abou-Rizk, 2021, [23]	Asia	Syrian	cross sectional	0–5 years	433	30.5
Theresa Jeremias, 2023, [25]		Syrian	cross sectional	0–2 years	215	42.0
Rima Rafiq El Kishawi, 2015, [13]		Palestinian	cross sectional	2–5 years	357	59.7
Leidman E, 2018 [24]		Bangladesh	cross sectional	0.5–5 years	269	47.9
Gideon Koren, 2019 [35]		Southern Tel Aviv	cross sectional	0–12 years	386	34.0
Philip Ndemwa, 2011 [27]	Africa	Kenya	cross sectional	0.5–5 years	410	42.6
Bisrat Birke Teketelew, 2023 [28]		Ethiopia	cross sectional	0.5–14 years	354	33.62
Oluwaremilekun G. Ajakaye, 2019 [26]		Nigeria	cross sectional	0–10 years	250	54.0
Yasin Jemal, 2017 [29]		Ethiopia	cross sectional	0.5–5 years	399	52.4
Irene Ule Ngole Sumbele, 2020 [12]		Cameroon	Cross sectional	0–3 years	378	84.0

## Prevalence of anemia in global refugee children

**Fig 2:**
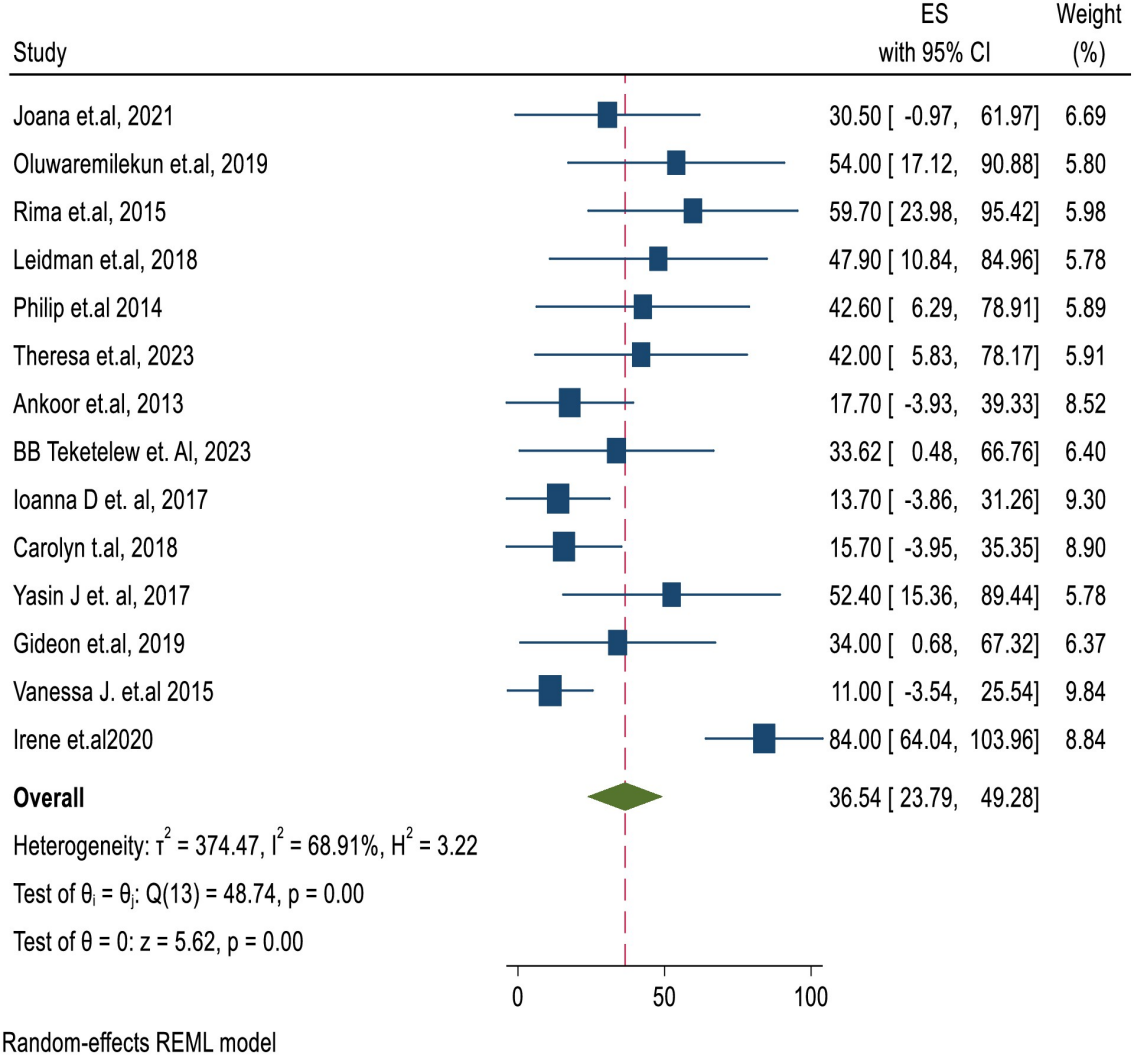
Forest plot showing global pooled prevalence of anemia among refugee children. The midpoint and the length of each segment indicates prevalence and a 95% confidence interval (CI), whereas the diamond shape shows the combined prevalence of all studies.

The pooled prevalence of anemia among refugee children of the world was 36.54% with (95% CI: 23.79, 49.28). There was moderate level of heterogeneity between the studies (I^2^ = 68.91, P<0.001) Fig 2.

### Subgroup analysis

The meta-analysis showed moderate level of heterogeneity between studies; hence subgroup analysis was performed to determine the source of heterogeneity. Based on the subgroup analysis, the highest pooled prevalence of anemia in refugee children was reported in African, it was 56.1%, whereas the lowest pooled prevalence of anemia was in N. America, it was 12.66%. The prevalence of anemia was highest (53.90) in studies done among under-five refugee children, while it was lower (15.1%) among under eighteen refugee children. The degree of heterogeneity among the studies included in the subgroup analysis was significant (P <0.05) Table 2.

**Table 2:** Subgroup analysis on prevalence of anemia among global refugee children.

Variables	subgroup	Number of studies	Prevalence %	I^2^%	P-value
Continent	Africa	5	56.1	62.11	<0.001
	Asia	5	41.81	68.91	<0.001
	Europe	2	15.29	31.81	0.028
	N. America	2	12.66	26.30	0.034
Age group	Under 5 years	7	53.90	45.93	<0.001
	Under 12 years	2	42.90	28.41	0.001
	Under 18 years	5	15.10	64.11	0.001
Publication year	2010–2014	2	25.71	25.01	0.027
	2015–2018	6	27.57	65.14	0.001
	2019–2023	6	48.58	59.13	<0.001

## Publication bias

Egger’s regression analysis was analyses to assess the potential publication bias of the studies. Based on the analysis, there was no evidence of potential publication bias (P = 0.826) Table 3.

**Table 3:** Egger’s test assessing publication bias for included studies.

Meta-es	Coefficient	Std. error	T	P> t	95% CI
Meta-se	2.26	1.41	1.60	0.135	(-0.82, 5.35)
Bias	4.65	20.73	0.22	0.826	(-40.50, 49.80)

## Discussion

Anemia in refugee setting due to living condition is a serious public health concern in the global population particularly in children [4]. Natural and human disasters particularly drought and conflict have the primary roles for people’s displacement and reside to the refugee. Due to anemia has serious concern in refugee children, this review aims to explore and estimate the pooled prevalence of anemia in the global refugee children.

According to this systemic review and meta-analysis, the pooled prevalence of anemia in refugee children was 36.54% with (95% CI: 23.79, 49.28). This indicates that one third of the refugee children are affected by the condition, which has a profound implication on their growth and development. According to WHO anemia public health concern, this review showed the global anemia prevalence in refugee children is at moderate public health issue. The WHO categorizes anemia prevalence as public health concern when it exceeds 20% [36], and in our review, the pooled prevalence of anemia exceeds that threshold. This highlights the need to early intervention and evaluation of anemia in refugee children. This moderate public health concern of anemia also forward message and calls for increased attention from the global health care organizations, stockholders, policymakers to focus on anemia intervention by assessing and controlling the risk factors of anemia in such vulnerable population.

In this review, we found that anemia prevalence is high in African refugee children 56.1% [19, 20, 30, 37]. With highest prevalence reported in Cameroon 84% [30] and the lowest prevalence in Ethiopia 33.62 [19]. Based on the continent the lowest prevalence of anemia was reported in N. American refugee children 12.66% [31, 32]. The difference in anemia prevalence might be due to the living condition, socioeconomic standard and health facility of the hosting country. In Asian countries the prevalence of anemia was ranging from 30.5% to 59.7% with pooled prevalence of 41.81% [13, 23–25]. Moreover, the prevalence of anemia in Europe was found to be 15.29% [34, 38]. As showed above, anemia was mild public health problem in Europe and N. America, while it was severe public health concern in Asian and African refugee children.

Anemia has multifactorial causes and vary widely throughout the world due to nutritional, socio-economic, environmental and genetic factors. Nutritional deficiencies, malaria, helminth infection [26, 39] and in some extents sickle cell diseases are the common causes for anemia in sub–Saharan Africa [40]. Genetic disorder, iron deficiency, and chronic diseases are the common causes in North Africa, Europe, and North America [5, 41]. The high global prevalence of anemia in refugee and displaced children is due to poverty, food insecurity, infectious diseases, maternal health problems, and poor sanitation. In order to understand the reasons behind the varying prevalence of anemia in refugee children, we are conducting a subgroup analysis to provide support and assess the causes and potential sources of heterogeneity.

The subgroup analysis revealed that the prevalence of anemia was varied among groups of continents, age range of the included study and publication year. Based on this, the highest prevalence of anemia was reported in African refugee children, whereas the lowest in N. America, which is discussed in the former paragraph. Children in low-income countries may have higher risk for malnutrition and communicable diseases. There are multiple reasons that can explain why malnourished children have greater rates of anemia. Slower metabolism due to adaptation for malnutrition, low iron availability due to acute malnutrition and decrease immune system in malnourished children leads suffering for infection and indirectly compete and block iron absorption are the comment mechanisms for wasting and anemia [42, 43]. The other common factor in low-income refugee countries for the development of anemia was diarrhea. Children with diarrhea were more likely to have anemia [44]. Diarrhea increases the loss of nutrients, which has an impact on children’s nutritional status. Following episodes of diarrhea, malabsorption, vomiting, and poor digestion are possible. It is also possible to experience frequent fluid and electrolyte loss as well as malabsorption of proteins, carbs, and other minerals [45].

Anemia was also highest in studies including only under five years of age which is 53.9% [13, 23, 24, 27, 29] and lower in studies including all children under the age of 18 years. This indirectly shows anemia can affect younger children with the age of under rapid growing. Children during rapid growth they requires high demands of iron especially for erythrocyte development [46], however, displaced and refugee children in low-income country are vulnerable to micronutrient deficiency, infection due to overcrowding and food aid dependency [47, 48]. Infection especially parasites can impair the absorption of available dietary iron and increase the onset of anemia [16]. Moreover, the sub group analysis showed anemia was coming more prevalent year to year, it was 25.7 at the end of 2014 and this increase to 48.58 up to 2023. The lower prevalence between 2010 to 2014 might be due to few studies were included to the meta-analysis from high prevalent countries, it was still a serious concern and needs particular focus in refugee children to reduce it.

Reducing anemia in developing countries and refugees such as in African context requires multifaced approach due to a diverse cause throughout the continent. Based on our review, we suggest that improving nutrition such as micronutrient supplementation, food fortification, and promoting dietary diversity particularly for vulnerable groups is the primary action for anemia reduction. Deworming and combating infection are also the other important actions for the reduction of anemia in such resource constricted area. Moreover, enhancing maternal and child health, improving sanitation in refugee and displaced setting has a paramount important in reduction and prevention of anemia.

This review has some limitations, including not to included studies published with all language. So, it will better to conduct review including published articles with all language and conduct an umbrella review to see the global effect of anemia in refugee children. Moreover, on the side of the scientific community, it is better to assess important nutritional elements such as micronutrient level beyond iron including; vitamins, zinc, and details on iron profiles.

## Conclusion

This study provides a general review of prevalence of anemia among refugee children of the world. the global prevalence of anemia among refugee children is found to be moderate public health problem with particularly high rates observed in African refugee children, indicating geographic concentration of this public health problem. This underscores the importance of tailoring interventions to the specific needs of different regions and populations. Intervention and prevention for should be focused especially for refugees found in low- and middle-income countries. The prevalence of anemia is more common among young children, suggesting that age-specific vulnerabilities must be addressed in any intervention strategy. Consequently, focused policies and strategies should prioritize the intervention especially for younger refugee children. The prevalence of anemia among refugee children is still challenge throughout the world. as the subgroup analysis showed, anemia was increasing time to time.

In light of these findings, it is clear that a more robust, multi-faceted approach is required to address anemia among refugee children. This includes improving nutritional support, enhancing healthcare access, and implementing targeted educational programs for both healthcare providers and refugee communities. The global health community must prioritize this issue, recognizing that the well-being of refugee children is integral to the broader goals of global health and equity. In conclusion, while the prevalence of anemia among refugee children presents a complex challenge, it is one that can and must be addressed through coordinated, sustained, and culturally sensitive interventions.

## Supporting information

S1 File Search terms on databases for anemia review. (DOCX)

S2 File JBI risk of bias quality assessment checklist and quality score. (DOCX)

S3 File PRISMA2020 Research checklist. (DOCX)

S4 File List of excluded articles with reason. (DOCX)

S5 File Summary of extracted data. (DOCX)

S6 File Included articles with full title. (DOCX)
