# Supplementary material for: Global prevalence of anemia in displaced and refugee children: A comprehensive systematic review and meta-analysis
Source: PLoS One. 2024 Nov 22;19(11):e0312905. doi: 10.1371/journal.pone.0312905 (PMC11584123; doi:10.1371/journal.pone.0312905)
Supplement: S1 File — (DOCX) [file pone.0312905.s001.docx]

**PubMed**

(((((prevalence) OR (magnitude)) OR (burden)) AND ((anemia) OR (iron deficiency anemia))) AND ((((((displacement) OR (refugee)) OR (refugee camp)) OR (evacuee)) OR (fugitive)) OR (expatriate))) AND ((children) OR (under five children)). **15/08/2024**

**EMBASE**

('prevalence'/exp OR prevalence OR 'magnitude'/exp OR magnitude OR 'burden'/exp OR burden) AND ('anemia'/exp OR anemia OR (('iron'/exp OR iron) AND ('deficiency'/exp OR deficiency) AND ('anemia'/exp OR anemia))) AND ('displacement'/exp OR displacement OR 'refugee'/exp OR refugee 'evacuee'/exp OR evacuee OR 'fugitive'/exp OR fugitive OR 'expatriate'/exp OR expatriate OR (('refugee'/exp OR refugee) AND ('camp'/exp OR camp))) AND ('children'/exp OR children OR (under AND five AND ('children'/exp OR children))). **17/08/2024**

**Scopus**

TITLE-ABS-KEY(((((prevalence) OR (magnitude)) OR (burden)) AND ((anemia) OR (iron deficiency anemia))) AND ((((((displacement) OR (refugee)) OR (refugee camp)) OR (evacuee)) OR (fugitive)) OR (expatriate))) AND ((children) OR (under five children)). **17/08/2024.**
