# Supplementary material for: Global prevalence of anemia in displaced and refugee children: A comprehensive systematic review and meta-analysis
Source: PLoS One. 2024 Nov 22;19(11):e0312905. doi: 10.1371/journal.pone.0312905 (PMC11584123; doi:10.1371/journal.pone.0312905)
Supplement: S2 File — (DOCX) [file pone.0312905.s002.docx]

| **S/N** | **Author, year of publication** | **Q1** | **Q2** | **Q3** | **Q4** | **Q5** | **Q6** | **Q7** | **Q8** | **Q9** | **Total score** | **Percent scored** | **Remark for eligibility** |
| --- | --- | --- | --- | --- | --- | --- | --- | --- | --- | --- | --- | --- | --- |
| 1 | Joana, et al, 2021 | Y | Y | Y | Y | Y | Y | Y | Y | U | 8 | 88.89 |  |
| 2 | Oluwaremilekun, et al,2019 | Y | Y | Y | Y | Y | Y | Y | Y | NA | 8 | 88.89 |  |
| 3 | Rima, et al, 2015 | Y | Y | Y | Y | Y | Y | Y | Y | Y | 9 | 100 |  |
| 4 | Leidman, et al, 2018 | Y | Y | Y | Y | Y | Y | Y | Y | U | 8 | 88.89 |  |
| 5 | Philip, et al, 2011 | Y | Y | Y | NA | Y | Y | Y | Y | Y | 8 | 88.89 |  |
| 6 | Theresa, et al, 2023 | Y | Y | NA | Y | Y | Y | Y | Y | U | 7 | 77.78 |  |
| 7 | Ankoor, et al, 2013 | Y | Y | Y | NA | Y | Y | Y | Y | Y | 8 | 88.89 |  |
| 8 | Bisrat, et al, 2023 | Y | Y | Y | Y | Y | Y | Y | Y | Y | 9 | 100 |  |
| 9 | Loanna, et al, 2017 | Y | Y | Y | Y | Y | Y | Y | Y | U | 8 | 88.89 |  |
| 10 | Carolyn, et al, 2018 | Y | y | Y | NA | Y | Y | Y | Y | U | 7 | 77.78 |  |
| 11 | Yasin, et al | Y | Y | Y | Y | Y | Y | Y | Y | Y | 9 | 100 |  |
| 12 | Gideon, et al, 2019 | y | y | y | y | y | y | y | Y | y | 9 | 100 |  |
| 13 | Vanessa, et al, 2015 | Y | Y | Y | NA | Y | Y | Y | Y | U | 7 | 77.78 |  |
| 14 | Irene, et al, 2020 | Y | Y | Y | U | Y | Y | Y | Y | NA | 7 | 77.78 |  |

**Key:** **Y**= Yes; **N**= Not, **U**= unclear, **NA**=Not appropriate

**Question codes:**

1. Was the sample frame appropriate to address the target population?

2. Were study participants sampled in an appropriate way?

3. Was the sample size adequate?

4. Were the study subjects and the setting described in detail?

5. Was the data analysis conducted with sufficient coverage of the identified sample?

6. Were valid methods used for the identification of the condition?

7. Was the condition measured in a standard, reliable way for all participants?

8. Was there appropriate statistical analysis?

9. was the response rate adequate, and if not, was the low response rate managed appropriately?
