# Supplementary material for: Global prevalence of anemia in displaced and refugee children: A comprehensive systematic review and meta-analysis
Source: PLoS One. 2024 Nov 22;19(11):e0312905. doi: 10.1371/journal.pone.0312905 (PMC11584123; doi:10.1371/journal.pone.0312905)
Supplement: S4 File — (DOCX) [file pone.0312905.s004.docx]

**Table3: List of excluded articles from the total search and not included in the final meta-analysis (N=164)**

| **S/N** | **Author Year** | **Articles title** | **Reason for exclusion** |
| --- | --- | --- | --- |
| 1 | [Christian Harkense](https://pubmed.ncbi.nlm.nih.gov/?term=Harkensee+C&cauthor_id=33783882) 2021 | Health needs of accompanied refugee and asylum-seeking children in a UK specialist clinic | Not related to the review objective and excluded by title and abstract |
| 2 | [Hind Khalid Sabeeh](https://pubmed.ncbi.nlm.nih.gov/?term=Sabeeh+HK&cauthor_id=35204934), 2022 | Iraq Is Moving Forward to Achieve Global Targets in Nutrition | Not related to the review objective and excluded by title and abstract |
| 3 | [Angela M D Sandel](https://pubmed.ncbi.nlm.nih.gov/?term=Sandell+AMD&cauthor_id=28657924) | Health Status and Anthropometric Changes in Resettled Refugee Children | Not related to the review objective and excluded by title and abstract |
| 4 | [Arzu Yazal Erdem](https://pubmed.ncbi.nlm.nih.gov/?term=Yazal+Erdem+A&cauthor_id=30706992), 2019 | Refugee children with beta-thalassemia in Turkey: Overview of demographic, socioeconomic, and medical characteristics | Not related to the review objective and excluded by title and abstract |
| 5 | [Laura Smock](https://pubmed.ncbi.nlm.nih.gov/?term=Smock+L&cauthor_id=29672357), 2019 | Refugee Children's Participation in the Women, Infants, and Children Supplemental Nutrition (WIC) Program in Massachusetts, 1998-2010 | Not related to the review objective and excluded by title and abstract |
| 6 | [Anum Saeedullah](https://pubmed.ncbi.nlm.nih.gov/?term=Saeedullah+A&cauthor_id=34578948) , 2021 | Nutritional Status of Adolescent Afghan Refugees Living in Peshawar, Pakistan | Not related to the review objective and excluded by title and abstract |
| 7 | [B Zur](https://pubmed.ncbi.nlm.nih.gov/?term=Zur+B&cauthor_id=26899228), 2016 | Increase in genetically determined anemia as a result of migration in Germany | Not related to the review objective and excluded by title and abstract |
| 8 | [Julie Kim](https://pubmed.ncbi.nlm.nih.gov/?term=Kim+J&cauthor_id=34714345), 2021 | Estimating the Burden of Child Undernutrition for Smaller Electoral Units in India | Not related to the review objective and excluded by title and abstract |
| 9 | [Christina D Lupone](https://pubmed.ncbi.nlm.nih.gov/?term=Lupone+CD&cauthor_id=30895418), 2020 | Lead Exposure in Newly Resettled Pediatric Refugees in Syracuse, NY | Not related to the review objective and excluded by title and abstract |
| 10 | [Najibah A Galadanci](https://pubmed.ncbi.nlm.nih.gov/?term=Galadanci+NA&cauthor_id=34486556), 2021 | Treatment-related Correlates of Growth in Children With Sickle Cell Disease in the DISPLACE Cohort | Not related to the review objective and excluded by title and abstract |
| 11 | [Ankoor Y Shah](https://pubmed.ncbi.nlm.nih.gov/?term=Shah+AY&cauthor_id=23828627), 2014 | Nutritional status of refugee children entering DeKalb County, Georgia | Not related to the review objective and excluded by title and abstract |
| 12 | [Caitlyn Lutfy](https://pubmed.ncbi.nlm.nih.gov/?term=Lutfy+C&cauthor_id=23430464), 2014 | Malnourished children in refugee camps and lack of connection with services after US resettlement | Not related to the review objective and excluded by title and abstract |
| 13 | [Oleg Bilukha](https://pubmed.ncbi.nlm.nih.gov/?term=Bilukha+O&cauthor_id=22073800), 2011 | Effects of multimicronutrient home fortification on anemia and growth in Bhutanese refugee children | Not related to the review objective and excluded by title and abstract |
| 14 | [Emily Esmaili](https://pubmed.ncbi.nlm.nih.gov/?term=Esmaili+E&cauthor_id=33972271), 2021 | Pediatric Refugee Health and Patterns of Health Care Utilization in Durham, North Carolina | Not related to the review objective and excluded by title and abstract |
| 15 | [Jee Hyun Rah](https://pubmed.ncbi.nlm.nih.gov/?term=Rah+JH&cauthor_id=22131547), 2021 | Program experience with micronutrient powders and current evidence | Not related to the review objective and excluded by title and abstract |
| 16 | [Lambed Tatah](https://pubmed.ncbi.nlm.nih.gov/?term=Tatah+L&cauthor_id=36506401), 2022 | Resilience in maternal and child nutrition outcomes in a refugee-hosting community in Cameroon: A quasi-experimental study | Not related to the review objective and excluded by title and abstract |
| 17 | [Sarah Adelman](https://pubmed.ncbi.nlm.nih.gov/?term=Adelman+S&cauthor_id=30926996), 2019 | School Feeding Reduces Anemia Prevalence in Adolescent Girls and Other Vulnerable Household Members in a Cluster Randomized Controlled Trial in Uganda | Not related to the review objective and excluded by title and abstract |
| 18 | [Idil Rana User](https://pubmed.ncbi.nlm.nih.gov/?term=User+IR&cauthor_id=31037325), 2019 | Common pediatric surgical diseases of refugee children: health around warzone | Not related to the review objective and excluded by title and abstract |
| 19 | [Katherine Yun](https://pubmed.ncbi.nlm.nih.gov/?term=Yun+K&cauthor_id=26562126), 2016 | Health Profiles of Newly Arrived Refugee Children in the United States, 2006-2012 | Not related to the review objective and excluded by title and abstract |
| 20 | [K Alnuaim](https://pubmed.ncbi.nlm.nih.gov/?term=Alnuaimi+K&cauthor_id=28542791), 2017 | Pregnancy outcomes among Syrian refugee and Jordanian women: a comparative study | Not related to the review objective and excluded by title and abstract |
| 21 | [Waqar Jeelani](https://pubmed.ncbi.nlm.nih.gov/?term=Jeelani+W&cauthor_id=33503121), 2020 | Nature and severity of dental malocclusion in children suffering from transfusion-dependent (-thalassemia major | Not related to the review objective and excluded by title and abstract |
| 22 | [Kerui Xu](https://pubmed.ncbi.nlm.nih.gov/?term=Xu+K&cauthor_id=30835394), 2018 | Common Diagnoses among Refugee Populations: Linked Results with Statewide Hospital Discharge Database | Not related to the review objective and excluded by title and abstract |
| 23 | [Katherine Tripp](https://pubmed.ncbi.nlm.nih.gov/?term=Tripp+K&cauthor_id=19476680), 2010 | Acceptability and use of iron and iron-alloy cooking pots: implications for anaemia control programmes | Not related to the review objective and excluded by title and abstract |
| 24 | [Masako Horino](https://pubmed.ncbi.nlm.nih.gov/?term=Horino+M&cauthor_id=33131324), 2020 | Dietary Inadequacy, Micronutrient Deficiencies, and Approaches to Preventing Poor Nutrition in the Gaza Strip | Not related to the review objective and excluded by title and abstract |
| 25 | [Melaku Tadege Engidaw](https://pubmed.ncbi.nlm.nih.gov/?term=Engidaw+MT&cauthor_id=30308060), 2018 | Anemia and associated factors among adolescent girls living in Aw-Barre refugee camp, Somali regional state, Southeast Ethiopia | Not related to the review objective and excluded by title and abstract |
| 26 | [L Zunino](https://pubmed.ncbi.nlm.nih.gov/?term=Zunino+L&cauthor_id=34511280), 2021 | Description of a migrant pediatric population visiting the Toulouse Children's Hospital emergency department | Not related to the review objective and excluded by title and abstract |
| 27 | [Laura Smock](https://pubmed.ncbi.nlm.nih.gov/?term=Smock+L&cauthor_id=30969273), 2020 | Recovery From Malnutrition Among Refugee Children Following Participation in the Special Supplemental Nutrition for Women, Infants, and Children (WIC) Program in Massachusetts, 1998-2010 | Not related to the review objective and excluded by title and abstract |
| 28 | [A A M Stellinga-Boelen](https://pubmed.ncbi.nlm.nih.gov/?term=Stellinga-Boelen+AA&cauthor_id=18030238), 2007 | Iron deficiency among children of asylum seekers in the Netherlands | Not related to the review objective and excluded by title and abstract |
| 29 | [Sarah Seifu](https://pubmed.ncbi.nlm.nih.gov/?term=Seifu+S&cauthor_id=30888565), 2020 | The Prevalence of Elevated Blood Lead Levels in Foreign-Born Refugee Children Upon Arrival to the U.S. and the Adequacy of Follow-up Treatment | Not related to the review objective and excluded by title and abstract |
| 30 | [Asad Mustafa Karim](https://pubmed.ncbi.nlm.nih.gov/?term=Karim+AM&cauthor_id=33939717), 2021 | Prevalence of clinical malaria and household characteristics of patients in tribal districts of Pakistan | Not related to the review objective and excluded by title and abstract |
| 31 | [Paul Oboth](https://pubmed.ncbi.nlm.nih.gov/?term=Oboth+P&cauthor_id=30935405), 2019 | Prevalence and clinical outcomes of Plasmodium falciparum and intestinal parasitic infections among children in Kiryandongo refugee camp, mid-Western Uganda: a cross sectional study | Not related to the review objective and excluded by title and abstract |
| 32 | [Adekunle D Adekile](https://pubmed.ncbi.nlm.nih.gov/?term=Adekile+AD&cauthor_id=31144996), 2019 | The Sub-Phenotypes of Sickle Cell Disease in Kuwait | Not related to the review objective and excluded by title and abstract |
| 33 | [Oleg O Bilukha](https://pubmed.ncbi.nlm.nih.gov/?term=Bilukha+OO&cauthor_id=25055188), 2014 | Nutritional status of women and child refugees from Syria-Jordan, April-May 2014 | Not related to the review objective and excluded by title and abstract |
| 34 | [Luana Santana Brito](https://pubmed.ncbi.nlm.nih.gov/?term=Brito+LS&cauthor_id=37283433), 2023 | Transitions experienced by mothers of children/adolescents with sickle cell disease in the context of the COVID-19 pandemic | Not related to the review objective and excluded by title and abstract |
| 35 | [Abdullah Sulieman Terkawi](https://pubmed.ncbi.nlm.nih.gov/?term=Terkawi+AS&cauthor_id=31143699), 2019 | Child and adolescent health in northwestern Syria: findings from Healthy-Syria 2017 study | Not related to the review objective and excluded by title and abstract |
| 36 | [Parminder S Suchdev](https://pubmed.ncbi.nlm.nih.gov/?term=Suchdev+PS&cauthor_id=32107773), 2020 | Home fortification of foods with multiple micronutrient powders for health and nutrition in children under two years of age | Not related to the review objective and excluded by title and abstract |
| 37 | [Salwa Massad](https://pubmed.ncbi.nlm.nih.gov/?term=Massad+S&cauthor_id=32864153) , 2020 | Micronutrient status of Palestinian school children following salt and flour fortification: a cross-sectional study | Not related to the review objective and excluded by title and abstract |
| 38 | [L Marquardt](https://pubmed.ncbi.nlm.nih.gov/?term=Marquardt+L&cauthor_id=26610271), 2016 | Health status and disease burden of unaccompanied asylum-seeking adolescents in Bielefeld, Germany: cross-sectional pilot study | Not related to the review objective and excluded by title and abstract |
| 39 | [Asad Mustafa Karim](https://pubmed.ncbi.nlm.nih.gov/?term=Karim+AM&cauthor_id=26809063), 2016 | Epidemiology and Clinical Burden of Malaria in the War-Torn Area, Orakzai Agency in Pakistan | Not related to the review objective and excluded by title and abstract |
| 40 | [Andrew J Seal](https://pubmed.ncbi.nlm.nih.gov/?term=Seal+AJ&cauthor_id=15795439), 2005 | Iron and vitamin A deficiency in long-term African refugees | Not related to the review objective and excluded by title and abstract |
| 41 | [D Hurst](https://pubmed.ncbi.nlm.nih.gov/?term=Hurst+D&cauthor_id=6842323), 1983 | Anemia and hemoglobinopathies in Southeast Asian refugee children | Not related to the review objective and excluded by title and abstract |
| 42 | [Paul L Geltman](https://pubmed.ncbi.nlm.nih.gov/?term=Geltman+PL&cauthor_id=31539488), 2019 | Trends in Elevated Blood Lead Levels Using 5 and 10 µg/dL Levels of Concern Among Refugee Children Resettled in Massachusetts, 1998-2015 | Not related to the review objective and excluded by title and abstract |
| 43 | [Najibah A Galadanci](https://pubmed.ncbi.nlm.nih.gov/?term=Galadanci+NA&cauthor_id=34966346), 2021 | Association Between Patent Foramen Ovale and Overt Ischemic Stroke in Children With Sickle Cell Disease | Not related to the review objective and excluded by title and abstract |
| 44 | [Tarissa Mitchell](https://pubmed.ncbi.nlm.nih.gov/?term=Mitchell+T&cauthor_id=22250021), 2012 | Lead poisoning in United States-bound refugee children: Thailand-Burma border, 2009 | Not related to the review objective and excluded by title and abstract |
| 45 | [M Laurence Noisette](https://pubmed.ncbi.nlm.nih.gov/?term=Noisette+ML&cauthor_id=33235147), 2021 | Changes in Care Delivery for Children With Sickle Cell Anemia During the COVID-19 Pandemic | Not related to the review objective and excluded by title and abstract |
| 46 | [Koichi Hashimoto](https://pubmed.ncbi.nlm.nih.gov/?term=Hashimoto+K&cauthor_id=37899541) , 2023 | Blood data trends of children in Fukushima after the Great East Japan Earthquake: Fukushima health management survey | Not related to the review objective and excluded by title and abstract |
| 47 | [Georgia A Paxton](https://pubmed.ncbi.nlm.nih.gov/?term=Paxton+GA&cauthor_id=22693599), 2012 | Post-arrival health screening in Karen refugees in Australia | Not related to the review objective and excluded by title and abstract |
| 48 | [Joana Abou-Rizk](https://pubmed.ncbi.nlm.nih.gov/?term=Abou-Rizk+J&cauthor_id=34842129), 2022 | Food insecurity, low dietary diversity and poor mental health among Syrian refugee mothers living in vulnerable areas of Greater Beirut, Lebanon | Not related to the review objective and excluded by title and abstract |
| 49 | [Bradley A Woodruff](https://pubmed.ncbi.nlm.nih.gov/?term=Woodruff+BA&cauthor_id=16480530), 2006 | Anaemia, iron status and vitamin A deficiency among adolescent refugees in Kenya and Nepal | Not related to the review objective and excluded by title and abstract |
| 50 | [Bradley Chen](https://pubmed.ncbi.nlm.nih.gov/?term=Chen+B&cauthor_id=27435004), 2016 | The impact of internal displacement on child mortality in post-earthquake Haiti: a difference-in-differences analysis | Not related to the review objective and excluded by title and abstract |
| 51 | [Philip Ndemwa](https://pubmed.ncbi.nlm.nih.gov/?term=Ndemwa+P&cauthor_id=22073802) , 2011 | Relationship of the availability of micronutrient powder with iron status and hemoglobin among women and children in the Kakuma Refugee Camp, Kenya | Not related to the review objective and excluded by title and abstract |
| 52 | [Tarissa Mitchell](https://pubmed.ncbi.nlm.nih.gov/?term=Mitchell+T&cauthor_id=29260657), 2018 | Impact of Enhanced Health Interventions for United States-Bound Refugees: Evaluating Best Practices in Migration Health | Not related to the review objective and excluded by title and abstract |
| 53 | [T Izutsu](https://pubmed.ncbi.nlm.nih.gov/?term=Izutsu+T&cauthor_id=16425652), 2005 | Nutritional and mental health status of Afghan refugee children in Peshawar, Pakistan: a descriptive study | Not related to the review objective and excluded by title and abstract |
| 54 | [Frank M Smithuis](https://pubmed.ncbi.nlm.nih.gov/?term=Smithuis+FM&cauthor_id=24119916), 2013 | The effect of insecticide-treated bed nets on the incidence and prevalence of malaria in children in an area of unstable seasonal transmission in western Myanmar | Not related to the review objective and excluded by title and abstract |
| 55 | [Sally Banfield](https://pubmed.ncbi.nlm.nih.gov/?term=Banfield+S&cauthor_id=22701664) , 2012 | Factors associated with the performance of a blood-based interferon-γ release assay in diagnosing tuberculosis | Not related to the review objective and excluded by title and abstract |
| 56 | [Carolyn Beukeboom](https://pubmed.ncbi.nlm.nih.gov/?term=Beukeboom+C&cauthor_id=29611017), 2018 | Prevalence of Nutritional Deficiencies Among Populations of Newly Arriving Government Assisted Refugee Children to Kitchener/Waterloo, Ontario, Canada | Not related to the review objective and excluded by title and abstract |
| 57 | [Najibah A Galadanci](https://pubmed.ncbi.nlm.nih.gov/?term=Galadanci+NA&cauthor_id=35417940), 2022 | Factors associated with left ventricular hypertrophy in children with sickle cell disease: results from the DISPLACE study | Not related to the review objective and excluded by title and abstract |
| 58 | [M A Drake](https://pubmed.ncbi.nlm.nih.gov/?term=Drake+MA&cauthor_id=1594741), 1992 | The nutritional status and dietary adequacy of single homeless women and their children in shelters | Not related to the review objective and excluded by title and abstract |
| 59 | [S M Moazzem Hossain](https://pubmed.ncbi.nlm.nih.gov/?term=Hossain+SM&cauthor_id=27872656), 2016 | Nutritional situation among Syrian refugees hosted in Iraq, Jordan, and Lebanon: cross sectional surveys | Not related to the review objective and excluded by title and abstract |
| 60 | [L Thielemans](https://pubmed.ncbi.nlm.nih.gov/?term=Thielemans+L&cauthor_id=29895274), 2018 | Indirect neonatal hyperbilirubinemia in hospitalized neonates on the Thai-Myanmar border: a review of neonatal medical records from 2009 to 2014 | Not related to the review objective and excluded by title and abstract |
| 61 | [Saskia de Pee](https://pubmed.ncbi.nlm.nih.gov/?term=de+Pee+S&cauthor_id=22073799), 2011 | Assessing the impact of micronutrient intervention programs implemented under special circumstances--meeting report | Not related to the review objective and excluded by title and abstract |
| 62 | [Ellen Andresen](https://pubmed.ncbi.nlm.nih.gov/?term=Andresen+E&cauthor_id=25121714), 2014 | Notes from the field: malnutrition and elevated mortality among refugees from South Sudan - Ethiopia, June-July 2014 | Not related to the review objective and excluded by title and abstract |
| 63 | [Vicki M Marsh](https://pubmed.ncbi.nlm.nih.gov/?term=Marsh+VM&cauthor_id=21797722), 2011 | All her children are born that way': gendered experiences of stigma in families affected by sickle cell disorder in rural Kenya | Not related to the review objective and excluded by title and abstract |
| 64 | [Burcu Fatma Belen](https://pubmed.ncbi.nlm.nih.gov/?term=Belen+BF&cauthor_id=26918459), 2016 | Frequency of neutropenia among Turkish and Syrian pediatric thalassemia patients under deferiprone monotherapy | Not related to the review objective and excluded by title and abstract |
| 65 | [Hala Ghattas](https://pubmed.ncbi.nlm.nih.gov/?term=Ghattas+H&cauthor_id=24739803), 2014 | Food insecurity among Iraqi refugees living in Lebanon, 10 years after the invasion of Iraq: data from a household survey | Not related to the review objective and excluded by title and abstract |
| 66 | [Hasan Ali Inal](https://pubmed.ncbi.nlm.nih.gov/?term=Inal+HA&cauthor_id=37393424), 2023 | Comparison of Perinatal Outcomes Between Syrian Refugees and Turkish Women in the Middle Anatolia Region of Turkey | Not related to the review objective and excluded by title and abstract |
| 67 | [Paula A Madrid](https://pubmed.ncbi.nlm.nih.gov/?term=Madrid+PA&cauthor_id=18935945), 2008 | Building integrated mental health and medical programs for vulnerable populations post-disaster: connecting children and families to a medical home | Not related to the review objective and excluded by title and abstract |
| 68 | [Sarah Style](https://pubmed.ncbi.nlm.nih.gov/?term=Style+S&cauthor_id=28591166), 2017 | Assessment of the effectiveness of a small quantity lipid-based nutrient supplement on reducing anaemia and stunting in refugee populations in the Horn of Africa: Secondary data analysis | Not related to the review objective and excluded by title and abstract |
| 69 | [Maurizio Brigotti](https://pubmed.ncbi.nlm.nih.gov/?term=Brigotti+M&cauthor_id=24068665), 2013 | Identification of TLR4 as the receptor that recognizes Shiga toxins in human neutrophils | Not related to the review objective and excluded by title and abstract |
| 70 | [Lucia De Francesch](https://pubmed.ncbi.nlm.nih.gov/?term=De+Franceschi+L&cauthor_id=30745303), 2019 | Access to emergency departments for acute events and identification of sickle cell disease in refugees | Not related to the review objective and excluded by title and abstract |
| 71 | [I M Khatib](https://pubmed.ncbi.nlm.nih.gov/?term=Khatib+IM&cauthor_id=20799573), 2010 | Nutritional interventions in refugee camps on Jordan's eastern border: assessment of status of vulnerable groups | Not related to the review objective and excluded by title and abstract |
| 72 | [Simon M Dyson](https://pubmed.ncbi.nlm.nih.gov/?term=Dyson+SM&cauthor_id=21375541), 2011 | Sickle cell, habitual dys-positions and fragile dispositions: young people with sickle cell at school | Not related to the review objective and excluded by title and abstract |
| 73 | [Laura Divens Zambrano](https://pubmed.ncbi.nlm.nih.gov/?term=Zambrano+LD&cauthor_id=32372751), 2020 | Clinical Sequelae Associated with Unresolved Tropical Splenomegaly in a Cohort of Recently Resettled Congolese Refugees in the United States-Multiple States, 2015-2018 | Not related to the review objective and excluded by title and abstract |
| 74 | [Toby Leslie](https://pubmed.ncbi.nlm.nih.gov/?term=Leslie+T&cauthor_id=20520804), 2010 | The impact of phenotypic and genotypic G6PD deficiency on risk of plasmodium vivax infection: a case-control study amongst Afghan refugees in Pakistan | Not related to the review objective and excluded by title and abstract |
| 75 | [O Monpierre](https://pubmed.ncbi.nlm.nih.gov/?term=Monpierre+O&cauthor_id=26860845), 2016 | Global health of unaccompanied refugee minors in Gironde (France) between 2011 and 2013 | Not related to the review objective and excluded by title and abstract |
| 76 | [Sarah Cherian](https://pubmed.ncbi.nlm.nih.gov/?term=Cherian+S&cauthor_id=19191761), 2009 | Helicobacter pylori, helminth infections and growth: a cross-sectional study in a high prevalence population | Not related to the review objective and excluded by title and abstract |
| 77 | [D Djokic](https://pubmed.ncbi.nlm.nih.gov/?term=Djokic+D&cauthor_id=21311633), [M B Drakulovic](https://pubmed.ncbi.nlm.nih.gov/?term=Drakulovic+MB&cauthor_id=21311633), 2010 | Risk factors associated with anemia among Serbian school-age children 7-14 years old: results of the first national health survey | Not related to the review objective and excluded by title and abstract |
| 78 | [Marios Loucas](https://pubmed.ncbi.nlm.nih.gov/?term=Loucas+M&cauthor_id=28719917), 2018 | Surgical Health Needs of Minor Refugees in Germany: A Cross-Sectional Study | Not related to the review objective and excluded by title and abstract |
| 79 | [Jill Benson](https://pubmed.ncbi.nlm.nih.gov/?term=Benson+J&cauthor_id=23469126), 2013 | Low vitamin B12 levels among newly-arrived refugees from Bhutan, Iran and Afghanistan: a multicentre Australian study | Not related to the review objective and excluded by title and abstract |
| 80 | [Matthew Burns](https://pubmed.ncbi.nlm.nih.gov/?term=Burns+M&cauthor_id=22855753), 2012 | Insecticide-treated plastic sheeting for emergency malaria prevention and shelter among displaced populations: an observational cohort study in a refugee setting in Sierra Leone | Not related to the review objective and excluded by title and abstract |
| 81 | [K Sonden](https://pubmed.ncbi.nlm.nih.gov/?term=Sonden+K&cauthor_id=25210980), 2014 | High incidence of Plasmodium vivax malaria in newly arrived Eritrean refugees in Sweden since May 2014 | Not related to the review objective and excluded by title and abstract |
| 82 | [Courtney D Thornburg](https://pubmed.ncbi.nlm.nih.gov/?term=Thornburg+CD&cauthor_id=29418070), 2018 | Children with sickle cell disease migrating to the United States from sub-Saharan Africa | Not related to the review objective and excluded by title and abstract |
| 83 | [Valentina D Mangano](https://pubmed.ncbi.nlm.nih.gov/?term=Mangano+VD&cauthor_id=25712976), 2015 | Novel Insights Into the Protective Role of Hemoglobin S and C Against Plasmodium falciparum Parasitemia | Not related to the review objective and excluded by title and abstract |
| 83 | [Deborah L McBride](https://pubmed.ncbi.nlm.nih.gov/?term=McBride+DL&cauthor_id=26718645), 2016 | Large Study of Health Issues for Newly Arrived Child Refugees | Not related to the review objective and excluded by title and abstract |
| 84 | [R E Brown](https://pubmed.ncbi.nlm.nih.gov/?term=Brown+RE&cauthor_id=4368728), 1974 | Field nutrition survey of Nigerian children in Ivory Coast refugee camps | Not related to the review objective and excluded by title and abstract |
| 85 | [G M Samuda](https://pubmed.ncbi.nlm.nih.gov/?term=Samuda+GM&cauthor_id=3395304), 1988 | Vietnamese child health in a Hong Kong closed camp | Not related to the review objective and excluded by title and abstract |
| 86 | [A M Gordon Jr](https://pubmed.ncbi.nlm.nih.gov/?term=Gordon+AM+Jr&cauthor_id=7064909), 1982 | Nutritional status of Cuban refugees: a field study on the health and nutriture of refugees processed at Opa Locka, Florida | Not related to the review objective and excluded by title and abstract |
| 87 | [Oleg Bilukha](https://pubmed.ncbi.nlm.nih.gov/?term=Bilukha+O&cauthor_id=22073800), 2011 | Effects of multimicronutrient home fortification on anemia and growth in Bhutanese refugee children | Not related to the review objective and excluded by title and abstract |
| 88 | [Katherine Tripp](https://pubmed.ncbi.nlm.nih.gov/?term=Tripp+K&cauthor_id=19476680), 2010 | Acceptability and use of iron and iron-alloy cooking pots: implications for anaemia control programmes | Not related to the review objective and excluded by title and abstract |
| 89 | [D M Parenti](https://pubmed.ncbi.nlm.nih.gov/?term=Parenti+DM&cauthor_id=3674256) , 1987 | Health status of Ethiopian refugees in the United States | Not related to the review objective and excluded by title and abstract |
| 90 | [Grace J Carroll](https://pubmed.ncbi.nlm.nih.gov/?term=Carroll+GJ&cauthor_id=28916577), 2017 | Evaluation of Nutrition Interventions in Children in Conflict Zones: A Narrative Review | Not related to the review objective and excluded by title and abstract |
| 92 | [Albertine Baauw](https://pubmed.ncbi.nlm.nih.gov/?term=Baauw+A&cauthor_id=31646192), 2019 | Health needs of refugee children identified on arrival in reception countries: a systematic review and meta-analysis | Not related to the review objective and excluded by title and abstract |
| 93 | [M J Bouma](https://pubmed.ncbi.nlm.nih.gov/?term=Bouma+MJ&cauthor_id=7747310), 1995 | Prevalence and clinical presentation of glucose-6-phosphate dehydrogenase deficiency in Pakistani Pathan and Afghan refugee communities in Pakistan; implications for the use of primaquine in regional malaria control programmes | Not related to the review objective and excluded by title and abstract |
| 94 | Abou-Rizk, 2021 | Anemia and nutritional status of syrian refugee mothers and their children under five years in greater Beirut, Lebanon | Duplicate removed |
| 95 | Ajakaye, 2020 | Prevalence and risk of malaria, anemia and malnutrition among children in IDPs camp in Edo State, Nigeria | Duplicate removed |
| 96 | Bilukha, 2011 | Effects of multimicronutrient home fortification on anemia and growth in Bhutanese refugee children | Duplicate removed |
| 97 | Darlan, 2018 | Correlation between iron deficiency anemia and intestinal parasitic infection in school-age children in Medan | Duplicate removed |
| 98 | El Kishawi, 2015 | Anemia among children aged 2-5 years in the Gaza Strip- Palestinian: a cross sectional study | Duplicate removed |
| 99 | Eshete, 2022 | Geographical pattern and associated factors of anemia among children aged 6–59 months in Ethiopia: Further analysis of Ethiopian demographic and health survey 2016 | Duplicate removed |
| 100 | GANDHI, 2022 | From Horror to Hope: Recognizing and Preventing the Health Impacts of War | Duplicate removed |
| 101 | Horino, 2023 | Triple Burden of Malnutrition among settled populations and Syrian and Palestinian refugees in Jordan | Duplicate removed |
| 102 | Hulland, 2021 | Anemia design effects in cluster surveys of women and young children in refugee settings | Duplicate removed |
| 103 | Jemal, 2017 | The magnitude and determinants of anaemia among refugee preschool children from the Kebribeyah refugee camp, Somali region, Ethiopia | Duplicate removed |
| 104 | Jeremias, 2023 | Anemia among Syrian Refugee Children Aged 6 to 23 Months Living in Greater Beirut, Lebanon, including the Voices of Mothers’ and Local Healthcare Staff: A Mixed-Methods Study | Duplicate removed |
| 105 | Leidman, 2018 | Acute malnutrition and anemia among Rohingya children in Kutupalong Camp, Bangladesh | Duplicate removed |
| 106 | Redditt, 2015 | Health status of newly arrived refugees in Toronto, Ont: Part 2: chronic diseases | Duplicate removed |
| 107 | Sumbele, 2020 | Burden of moderate to severe anaemia and severe stunting in children< 3 years in conflict-hit Mount Cameroon: a community based descriptive cross-sectional study | Duplicate removed |
| 108 | Teketelew, 2023 | Anemia and associated factors among internally displaced children at Debark refugee camp, North Gondar, Northwest Ethiopia | Duplicate removed |
| 109 | [Jee Hyun Rah](https://pubmed.ncbi.nlm.nih.gov/?term=Rah+JH&cauthor_id=22131547), 2021 | Program experience with micronutrient powders and current evidence | Duplicate removed |
| 110 | [Lambed Tatah](https://pubmed.ncbi.nlm.nih.gov/?term=Tatah+L&cauthor_id=36506401), 2022 | Resilience in maternal and child nutrition outcomes in a refugee-hosting community in Cameroon: A quasi-experimental study | Duplicate removed |
| 111 | [Sarah Adelman](https://pubmed.ncbi.nlm.nih.gov/?term=Adelman+S&cauthor_id=30926996), 2019 | School Feeding Reduces Anemia Prevalence in Adolescent Girls and Other Vulnerable Household Members in a Cluster Randomized Controlled Trial in Uganda | Duplicate removed |
| 112 | [Idil Rana User](https://pubmed.ncbi.nlm.nih.gov/?term=User+IR&cauthor_id=31037325), 2019 | Common pediatric surgical diseases of refugee children: health around warzone | Duplicate removed |
| 113 | [Katherine Yun](https://pubmed.ncbi.nlm.nih.gov/?term=Yun+K&cauthor_id=26562126), 2016 | Health Profiles of Newly Arrived Refugee Children in the United States, 2006-2012 | Duplicate removed |
| 114 | [K Alnuaim](https://pubmed.ncbi.nlm.nih.gov/?term=Alnuaimi+K&cauthor_id=28542791), 2017 | Pregnancy outcomes among Syrian refugee and Jordanian women: a comparative study | Duplicate removed |
| 115 | [Waqar Jeelani](https://pubmed.ncbi.nlm.nih.gov/?term=Jeelani+W&cauthor_id=33503121), 2020 | Nature and severity of dental malocclusion in children suffering from transfusion-dependent (-thalassemia major | Duplicate removed |
| 116 | [Jee Hyun Rah](https://pubmed.ncbi.nlm.nih.gov/?term=Rah+JH&cauthor_id=22131547), 2021 | Program experience with micronutrient powders and current evidence | Duplicate removed |
| 117 | [Marios Loucas](https://pubmed.ncbi.nlm.nih.gov/?term=Loucas+M&cauthor_id=28719917), 2018 | Surgical Health Needs of Minor Refugees in Germany: A Cross-Sectional Study | Duplicate removed |
| 118 | [Jill Benson](https://pubmed.ncbi.nlm.nih.gov/?term=Benson+J&cauthor_id=23469126), 2013 | Low vitamin B12 levels among newly-arrived refugees from Bhutan, Iran and Afghanistan: a multicentre Australian study | Duplicate removed |
| 119 | [Matthew Burns](https://pubmed.ncbi.nlm.nih.gov/?term=Burns+M&cauthor_id=22855753), 2012 | Insecticide-treated plastic sheeting for emergency malaria prevention and shelter among displaced populations: an observational cohort study in a refugee setting in Sierra Leone | Duplicate removed |
| 120 | [K Sonden](https://pubmed.ncbi.nlm.nih.gov/?term=Sonden+K&cauthor_id=25210980), 2014 | High incidence of Plasmodium vivax malaria in newly arrived Eritrean refugees in Sweden since May 2014 | Duplicate removed |
| 121 | [Courtney D Thornburg](https://pubmed.ncbi.nlm.nih.gov/?term=Thornburg+CD&cauthor_id=29418070), 2018 | Children with sickle cell disease migrating to the United States from sub-Saharan Africa | Duplicate removed |
| 122 | [Valentina D Mangano](https://pubmed.ncbi.nlm.nih.gov/?term=Mangano+VD&cauthor_id=25712976), 2015 | Novel Insights Into the Protective Role of Hemoglobin S and C Against Plasmodium falciparum Parasitemia | Duplicate removed |
| 123 | [Deborah L McBride](https://pubmed.ncbi.nlm.nih.gov/?term=McBride+DL&cauthor_id=26718645), 2016 | Large Study of Health Issues for Newly Arrived Child Refugees | Duplicate removed |
| 124 | [R E Brown](https://pubmed.ncbi.nlm.nih.gov/?term=Brown+RE&cauthor_id=4368728), 1974 | Field nutrition survey of Nigerian children in Ivory Coast refugee camps | Duplicate removed |
| 125 | [G M Samuda](https://pubmed.ncbi.nlm.nih.gov/?term=Samuda+GM&cauthor_id=3395304), 1988 | Vietnamese child health in a Hong Kong closed camp | Duplicate removed |
| 126 | [Marios Loucas](https://pubmed.ncbi.nlm.nih.gov/?term=Loucas+M&cauthor_id=28719917), 2018 | Surgical Health Needs of Minor Refugees in Germany: A Cross-Sectional Study | Duplicate removed |
| 127 | [Jill Benson](https://pubmed.ncbi.nlm.nih.gov/?term=Benson+J&cauthor_id=23469126), 2013 | Low vitamin B12 levels among newly-arrived refugees from Bhutan, Iran and Afghanistan: a multicentre Australian study | Duplicate removed |
| 128 | [Matthew Burns](https://pubmed.ncbi.nlm.nih.gov/?term=Burns+M&cauthor_id=22855753), 2012 | Insecticide-treated plastic sheeting for emergency malaria prevention and shelter among displaced populations: an observational cohort study in a refugee setting in Sierra Leone | Duplicate removed |
| 129 | [K Sonden](https://pubmed.ncbi.nlm.nih.gov/?term=Sonden+K&cauthor_id=25210980), 2014 | High incidence of Plasmodium vivax malaria in newly arrived Eritrean refugees in Sweden since May 2014 | Duplicate removed |
| 130 | [Georgia A Paxton](https://pubmed.ncbi.nlm.nih.gov/?term=Paxton+GA&cauthor_id=22693599), 2012 | Post-arrival health screening in Karen refugees in Australia | Duplicate removed |
| 131 | [Joana Abou-Rizk](https://pubmed.ncbi.nlm.nih.gov/?term=Abou-Rizk+J&cauthor_id=34842129), 2022 | Food insecurity, low dietary diversity and poor mental health among Syrian refugee mothers living in vulnerable areas of Greater Beirut, Lebanon | Duplicate removed |
| 132 | [Bradley A Woodruff](https://pubmed.ncbi.nlm.nih.gov/?term=Woodruff+BA&cauthor_id=16480530), 2006 | Anaemia, iron status and vitamin A deficiency among adolescent refugees in Kenya and Nepal | Duplicate removed |
| 133 | [Bradley Chen](https://pubmed.ncbi.nlm.nih.gov/?term=Chen+B&cauthor_id=27435004), 2016 | The impact of internal displacement on child mortality in post-earthquake Haiti: a difference-in-differences analysis | Duplicate removed |
| 134 | [Philip Ndemwa](https://pubmed.ncbi.nlm.nih.gov/?term=Ndemwa+P&cauthor_id=22073802) , 2011 | Relationship of the availability of micronutrient powder with iron status and hemoglobin among women and children in the Kakuma Refugee Camp, Kenya | Duplicate removed |
| 135 | [Tarissa Mitchell](https://pubmed.ncbi.nlm.nih.gov/?term=Mitchell+T&cauthor_id=29260657), 2018 | Impact of Enhanced Health Interventions for United States-Bound Refugees: Evaluating Best Practices in Migration Health | Duplicate removed |
| 136 | [T Izutsu](https://pubmed.ncbi.nlm.nih.gov/?term=Izutsu+T&cauthor_id=16425652), 2005 | Nutritional and mental health status of Afghan refugee children in Peshawar, Pakistan: a descriptive study | Duplicate removed |
| 137 | [Frank M Smithuis](https://pubmed.ncbi.nlm.nih.gov/?term=Smithuis+FM&cauthor_id=24119916), 2013 | The effect of insecticide-treated bed nets on the incidence and prevalence of malaria in children in an area of unstable seasonal transmission in western Myanmar | Duplicate removed |
| 138 | [Sally Banfield](https://pubmed.ncbi.nlm.nih.gov/?term=Banfield+S&cauthor_id=22701664) , 2012 | Factors associated with the performance of a blood-based interferon-γ release assay in diagnosing tuberculosis | Duplicate removed |
| 139 | [Carolyn Beukeboom](https://pubmed.ncbi.nlm.nih.gov/?term=Beukeboom+C&cauthor_id=29611017), 2018 | Prevalence of Nutritional Deficiencies Among Populations of Newly Arriving Government Assisted Refugee Children to Kitchener/Waterloo, Ontario, Canada | Duplicate removed |
| 140 | [Najibah A Galadanci](https://pubmed.ncbi.nlm.nih.gov/?term=Galadanci+NA&cauthor_id=35417940), 2022 | Factors associated with left ventricular hypertrophy in children with sickle cell disease: results from the DISPLACE study | Duplicate removed |
| 141 | [M A Drake](https://pubmed.ncbi.nlm.nih.gov/?term=Drake+MA&cauthor_id=1594741), 1992 | The nutritional status and dietary adequacy of single homeless women and their children in shelters | Duplicate removed |
| 142 | [S M Moazzem Hossain](https://pubmed.ncbi.nlm.nih.gov/?term=Hossain+SM&cauthor_id=27872656), 2016 | Nutritional situation among Syrian refugees hosted in Iraq, Jordan, and Lebanon: cross sectional surveys | Duplicate removed |
| 143 | [L Thielemans](https://pubmed.ncbi.nlm.nih.gov/?term=Thielemans+L&cauthor_id=29895274), 2018 | Indirect neonatal hyperbilirubinemia in hospitalized neonates on the Thai-Myanmar border: a review of neonatal medical records from 2009 to 2014 | Duplicate removed |
| 144 | [Saskia de Pee](https://pubmed.ncbi.nlm.nih.gov/?term=de+Pee+S&cauthor_id=22073799), 2011 | Assessing the impact of micronutrient intervention programs implemented under special circumstances--meeting report | Duplicate removed |
| 145 | [Ellen Andresen](https://pubmed.ncbi.nlm.nih.gov/?term=Andresen+E&cauthor_id=25121714), 2014 | Notes from the field: malnutrition and elevated mortality among refugees from South Sudan - Ethiopia, June-July 2014 | Duplicate removed |
| 146 | [Vicki M Marsh](https://pubmed.ncbi.nlm.nih.gov/?term=Marsh+VM&cauthor_id=21797722), 2011 | All her children are born that way': gendered experiences of stigma in families affected by sickle cell disorder in rural Kenya | Duplicate removed |
| 147 | [Burcu Fatma Belen](https://pubmed.ncbi.nlm.nih.gov/?term=Belen+BF&cauthor_id=26918459), 2016 | Frequency of neutropenia among Turkish and Syrian pediatric thalassemia patients under deferiprone monotherapy | Duplicate removed |
| 148 | [Hala Ghattas](https://pubmed.ncbi.nlm.nih.gov/?term=Ghattas+H&cauthor_id=24739803), 2014 | Food insecurity among Iraqi refugees living in Lebanon, 10 years after the invasion of Iraq: data from a household survey | Duplicate removed |
| 149 | [Hasan Ali Inal](https://pubmed.ncbi.nlm.nih.gov/?term=Inal+HA&cauthor_id=37393424), 2023 | Comparison of Perinatal Outcomes Between Syrian Refugees and Turkish Women in the Middle Anatolia Region of Turkey | Duplicate removed |
| 150 | [Paula A Madrid](https://pubmed.ncbi.nlm.nih.gov/?term=Madrid+PA&cauthor_id=18935945), 2008 | Building integrated mental health and medical programs for vulnerable populations post-disaster: connecting children and families to a medical home | Duplicate removed |
| 151 | [Sarah Style](https://pubmed.ncbi.nlm.nih.gov/?term=Style+S&cauthor_id=28591166), 2017 | Assessment of the effectiveness of a small quantity lipid-based nutrient supplement on reducing anaemia and stunting in refugee populations in the Horn of Africa: Secondary data analysis | Duplicate removed |
| 152 | [Maurizio Brigotti](https://pubmed.ncbi.nlm.nih.gov/?term=Brigotti+M&cauthor_id=24068665), 2013 | Identification of TLR4 as the receptor that recognizes Shiga toxins in human neutrophils | Duplicate removed |
| 153 | [Georgia A Paxton](https://pubmed.ncbi.nlm.nih.gov/?term=Paxton+GA&cauthor_id=22693599), 2012 | Post-arrival health screening in Karen refugees in Australia | Duplicate removed |
| 154 | [Alexandra Jablonka](https://pubmed.ncbi.nlm.nih.gov/?term=Jablonka+A&cauthor_id=29582203) 2015 | [Healthcare utilization in a large cohort of asylum seekers entering Western Europe in 2015](https://www.mdpi.com/1660-4601/15/10/2163) | Articles excluded due to containing ambiguous prevalence report, not fulfill the inclusion criteria |
| 155 | [Ariel Kay](https://pubmed.ncbi.nlm.nih.gov/?term=Kay+A&cauthor_id=31798995) 2019 | [The burden of anaemia among displaced women and children in refugee settings worldwide, 2013–2016](https://gh.bmj.com/content/4/6/e001837.abstract) | prevalence report, not fulfill the inclusion criteria |
| 156 | [Nada AbuKishk](https://pubmed.ncbi.nlm.nih.gov/?term=AbuKishk+N&cauthor_id=32967866) 2020 | [Anaemia prevalence in children newly registered at UNRWA schools: a cross-sectional study](https://bmjopen.bmj.com/content/10/9/e034705.abstract) | prevalence report, not fulfill the inclusion criteria |
| 157 | [Katherine Yun](https://pubmed.ncbi.nlm.nih.gov/?term=Yun+K&cauthor_id=26562126) 2016 | [Help-seeking behavior and health care navigation by Bhutanese refugees](https://link.springer.com/article/10.1007/s10900-015-0126-x) | prevalence report, not fulfill the inclusion criteria |
| 158 | Abou-Rizk J. 2021 | [Anemia and nutritional status of Syrian refugee mothers and their children under five years in greater Beirut, Lebanon](https://www.mdpi.com/1660-4601/18/13/6894) | prevalence report, not fulfill the inclusion criteria |
| 159 | Abu Kishk N. 2019 | [Prevalence of anaemia in children newly registered at UNRWA schools: a cross-sectional study](https://www.thelancet.com/journals/lancet/article/PIIS0140-6736(19)30621-X/abstract) | prevalence report, not fulfill the inclusion criteria |
| 160 | AbuKishk N. 2020 | [Anaemia prevalence in children newly registered at UNRWA schools: a cross-sectional study](https://bmjopen.bmj.com/content/10/9/e034705.abstract) | Duplicate and ambiguous sample size |
| 161 | [Katherine Tripp](https://pubmed.ncbi.nlm.nih.gov/?term=Tripp+K&cauthor_id=19476680) 2010 | [Acceptability and use of iron and iron-alloy cooking pots: implications for anaemia control programmes](https://www.cambridge.org/core/journals/public-health-nutrition/article/acceptability-and-use-of-iron-and-ironalloy-cooking-pots-implications-for-anaemia-control-programmes/2F39A0429559F24CF6FE0A75D1B7C8AD) | prevalence report, not fulfill the inclusion criteria |
| 162 | Zur B. 2016 | Increase in genetically determined anemia as a result of migration in Germany | prevalence report, not fulfill the inclusion criteria |
| 163 | Terkawi AS 2019 | Child and adolescent health in northwestern Syria: findings from Healthy-Syria 2017 study. Avicenna J Med. 2019 Apr-Jun;9(2):61-74 | prevalence report, not fulfill the inclusion criteria |
| 164 | Hala Ghattas 2014 | Food insecurity among Iraqi refugees living in Lebanon, 10 years after the invasion of Iraq: data from a household survey | Article excluded due to reported too small sample size |
