# Supplementary material for: Global prevalence of anemia in displaced and refugee children: A comprehensive systematic review and meta-analysis
Source: PLoS One. 2024 Nov 22;19(11):e0312905. doi: 10.1371/journal.pone.0312905 (PMC11584123; doi:10.1371/journal.pone.0312905)
Supplement: S5 File — (DOCX) [file pone.0312905.s005.docx]

**Table4: Summary characteristics of articles included in the systematic review (N = 14)**

| **First authors, year** | **Continent** | **Country** | **Study design** | **Age range** | **Sample size** | **prevalence%** | **Data extracted by** | **Date of data extraction** |
| --- | --- | --- | --- | --- | --- | --- | --- | --- |
| Carolyn Beukeboom, 2018 | N. America | Canada | retrospective | 0-16 years | 356 | 15.7 | Teketelew BB. and Tamir M. | Mar. 16, 2024 |
| Vanessa J. Redditt, 2015 |  | Toronto | retrospective | 0-15 years | 1063 | 11.0 | Teketelew BB. and Berta DM. | Mar16, 2024 |
| Ankoor Y. Shah, 2013 | Europe | Georgia | retrospective | 0-18 years | 555 | 17.7 | Cherie N. and Angelo AA. | Mar 23, 2024 |
| Ioanna D. Pavlopoulou, 2017, |  | Greece | cross sectional | 1-14 years | 300 | 13.7 | Chane E. and Cherie N | Mar 23, 2024 |
| Joana Abou-Rizk, 2021, | Asia | Syrian | cross sectional | 0-5 years | 433 | 30.5 | Tamir M. and Nigus M. | Mar 20, 2024 |
| Theresa Jeremias, 2023, |  | Syrian | cross sectional | 0-2 years | 215 | 42.0 | Mulatie Z. and Berta DM. | Apr 3, 2024 |
| Rima Rafiq El Kishawi, 2015, |  | Palestinian | cross sectional | 2-5 years | 357 | 59.7 | Teketelew BB. and Chane E. | Mar 27, 2024 |
| Leidman E, 2018 |  | Bangladesh | cross sectional | 0.5-5 years | 269 | 47.9 | Teketelew BB. And Chane E. | Apr 3, 2024 |
| Gideon Koren, 2019 |  | Southern Tel Aviv | cross sectional | 0-12 years | 386 | 34.0 | Cherie N. and Angelo AA | Apr 3, 2024 |
| Philip Ndemwa, 2011 | Africa | Kenya | cross sectional | 0.5-5 years | 410 | 42.6 | Tamir M. and Nigus M. | Mar 27, 2024 |
| Bisrat Birke Teketelew, 2023 |  | Ethiopia | cross sectional | 0.5-14 years | 354 | 33.62 | Chane E. and Cherie N | Apr 4, 2024 |
| Oluwaremilekun G. Ajakaye, 2019 |  | Nigeria | cross sectional | 0-10 years | 250 | 54.0 | Teketelew BB. Tamir M. | Apr 6, 2024 |
| Yasin Jemal, 2017 |  | Ethiopia | cross sectional | 0.5-5 years | 399 | 52.4 | Mulatie Z. and Berta DM. | Mar 29, 2024 |
| Irene Ule Ngole Sumbele, 2020 |  | Cameroon | Cross sectional | 0-3 years | 378 | 84.0 | Cherie N. and Angelo AA | Apr 6, 2024 |
