# Supplementary material for: Global prevalence of anemia in displaced and refugee children: A comprehensive systematic review and meta-analysis
Source: PLoS One. 2024 Nov 22;19(11):e0312905. doi: 10.1371/journal.pone.0312905 (PMC11584123; doi:10.1371/journal.pone.0312905)
Supplement: S6 File — (DOCX) [file pone.0312905.s006.docx]

**Table 5. List of included articles in the final systemic review and meta-analysis**

| **S/N** | **Author Year** | **Articles with full title** |
| --- | --- | --- |
| 1 | Carolyn Beukeboom, 2018 | Prevalence of nutritional deficiencies among populations of newly arriving government assisted refugee children to Kitchener/Waterloo, Ontario, Canada. |
| 2 | Vanessa J. Redditt | Health status of newly arrived refugees in Toronto, Ont: Part 2: chronic diseases |
| 3 | Ankoor Y. Shah, 2013 | Nutritional status of refugee children entering DeKalb County, Georgia |
| 4 | Ioanna D. Pavlopoulou, 2017 | Clinical and laboratory evaluation of new immigrant and refugee children arriving in Greece |
| 5 | Joana Abou-Rizk, 2021 | Anemia and nutritional status of syrian refugee mothers and their children under five years in greater Beirut, Lebanon |
| 6 | Theresa Jeremias, 2023 | Anemia among Syrian Refugee Children Aged 6 to 23 Months Living in Greater Beirut, Lebanon, including the Voices of Mothers’ and Local Healthcare Staff: A Mixed-Methods Study |
| 7 | Rima Rafiq El Kishawi, 2015, | Anemia among children aged 2–5 years in the Gaza Strip-Palestinian: a cross sectional study |
| 8 | Leidman E, 2018 | Acute malnutrition and anemia among Rohingya children in Kutupalong Camp, Bangladesh |
| 9 | Gideon Koren, 2019 | The prevalence of iron deficiency anemia among African asylum-seeking children residing in Tel Aviv |
| 10 | Philip Ndemwa, 2011 | Relationship of the availability of micronutrient powder with iron status and hemoglobin among women and children in the Kakuma Refugee Camp, Kenya. |
| 11 | Bisrat Birke Teketelew, 2023 | Anemia and associated factors among internally displaced children at Debark refugee camp, North Gondar, Northwest Ethiopia. |
| 12 | Oluwaremilekun G. Ajakaye, 2019 | Prevalence and risk of malaria, anemia and malnutrition among children in IDPs camp in Edo State, Nigeria |
| 13 | Yasin Jemal, 2017 | The magnitude and determinants of anaemia among refugee preschool children from the Kebribeyah refugee camp, Somali region, Ethiopia. |
| 14 | Irene Ule Ngole Sumbele, 2020 | Burden of moderate to severe anaemia and severe stunting in children < 3 years in conflict-hit Mount Cameroon: a community based descriptive cross-sectional study. |
